# Perinatal compassion focused therapy for mothers with mental health difficulties: a study protocol for a multisite and mixed methods feasibility and acceptability study

**DOI:** 10.3389/fpsyt.2025.1681673

**Published:** 2025-12-11

**Authors:** Leah A. Millard-Brewer, Debbie M. Smith, Ming Wai Wan, Anja Wittkowski

**Affiliations:** 1 Division of Psychology and Mental Health, School of Health Sciences, The University of Manchester, Manchester, United Kingdom; 2 Perinatal Mental Health and Parenting (PRIME) Research Unit, Greater Manchester Mental Health National Health Service (NHS) Foundation Trust, Manchester, United Kingdom; 3 Manchester Academic Health Science Centre, The University of Manchester, Manchester, United Kingdom

**Keywords:** compassionate, maternal mental health, self-compassion, psychological therapy, parent-infant relationship, women

## Abstract

**Introduction:**

Mental health difficulties are a common complication affecting 1 in 5 women during the perinatal period (from conception up to two years after childbirth). As well as affecting the wellbeing of the mother, perinatal mental health difficulties can contribute to adverse long-term outcomes for the infant by disrupting the mother-infant relationship. Effective interventions are needed to enhance maternal and infant wellbeing during the perinatal period. Perinatal compassion focused therapy (P-CFT) is a promising intervention that has been adapted for perinatal mental health difficulties, and which may also help enhance the mother-infant relationship through its focus on generating compassionate and empathic cognitions of self and others. While CFT is in use in perinatal clinical settings, few studies exist that explore its potential benefits. The aims of this study were to explore the feasibility of recruitment, to determine the suitability of the outcome measures, to assess the acceptability of P-CFT through qualitative evaluation, and to explore the effects of P-CFT on compassion-based outcomes, wellbeing, and parent-infant interactions.

**Methods:**

The multisite feasibility and acceptability study will adopt a single pre-post, quasi-experimental study design comprising three components using a sample of service users attending P-CFT groups. Component 1 is a feasibility study measuring maternal self-report questionnaire outcomes (e.g., self-compassion, postpartum bonding) at baseline, post-intervention and six-month follow-up, and recruitment and retention rates. Component 2 will evaluate mother-infant interactions through the coding of video observations from baseline to post-intervention. Component 3 will entail qualitative semi-structured interviews to explore the acceptability of the intervention. Data will be analysed using statistical methods of analysis for quantitative data (Component 1 and Component 2) and reflexive thematic analysis for qualitative data (Component 3).

**Discussion:**

To the authors’ knowledge, this study will be the first multisite feasibility and acceptability study of perinatal compassion focused therapy offered to mothers and birthing parents in specialist perinatal mental health services in England’s National Health Service (NHS). This study will identify any necessary refinements for future studies and considerations for good practice in this specific population and clinical setting, as well as the intervention itself.

## Introduction

Perinatal mental health (PMH) difficulties (mental disorders occurring during pregnancy or in the two years after childbirth) are the most prevalent complications of the childbearing period, experienced by approximately 10-20% of women (1) Thus, PMH difficulties constitute a serious public health issue. In women, PMH disorders are associated with distressing symptoms, poor functioning, increased morbidity and they are a leading cause of maternal death (2–4). PMH difficulties are also linked to obstetric pregnancy complications (e.g., low birthweight, pre-term birth) as well as poorer long-term outcomes for children in terms of their cognitive, social and emotional development (2, 5–7). Given the perinatal period is a critical time for maternal and infant mental health, and the long-term health of both, it is important to find ways of supporting and improving their well-being in the perinatal phase.

The National Institute for Health and Care Excellence (8) guidelines advocate for psychological interventions for PMH difficulties to address the mother-infant relationship. However, it is unclear if and/or how the current NICE-recommended interventions for perinatal women (cognitive behavioural therapy/CBT; interpersonal therapy/IPT) can improve the dyadic relationship (9). In England and Wales, perinatal mental health community teams (P-CMHTs) offer alternative psychological interventions that appear to be beneficial in improving both PMH difficulties and the parent-infant relationship (8).

Given the challenges of pregnancy and post-partum life, psychotherapeutic interventions such as compassion focused therapy (CFT) that incorporate self-compassion may be of particular benefit to a mother’s well-being and the parent-infant relationship (10, 11). The therapy can be delivered to service users in either a group or an individual format. According to Cree (10), high levels of self-criticism and associated low levels of self-compassion are common in mothers experiencing mental health problems, and these difficulties are associated with low wellbeing, problems in perceived bonding, and the parent-infant relationship (12). Underpinned by several psychological approaches (e.g., social, evolutionary, neuroscience), Gilbert’s (13, 14) compassion focused therapy (CFT) facilitates self-compassion in individuals using psychoeducation (e.g., imbalance of the emotional regulatory systems) and experiential compassion-focused exercises (e.g., compassionate voice, compassion letter writing). Reviews of CFT and related mindfulness-based interventions indicated that CFT led to generally positive outcomes in relation to self-compassion and mental health in clinical and non-clinical samples (15–19).

In our systematic review and meta-analysis of 15 randomised studies on people experiencing mental health difficulties, interventions based on Gilbert’s (13, 14) CFT model (primarily group therapy) was associated with significant improvements in compassion-based outcomes, such as levels of self-compassion and self-reassurance and clinical symptomology (e.g., depression, disordered eating (18). Furthermore, high levels of acceptability of group CFT to service users across various clinical groups were reported in a review and meta-synthesis of 12 studies of Gilbert’s (13, 14) model of CFT (20).

Based on Gilbert’s model of CFT, Michelle Cree (11) developed an adaptation in the context of parent-infant interactions called *Perinatal Compassion Focused Therapy* (P-CFT). This adaptation aims to promote the formation of a secure attachment between mother and infant (10). It addresses how the emotional systems can be affected during pregnancy, helping mothers to understand the exacerbation of shame and self-criticism during this period as well as emotional distress in general. This adaptation of P-CFT is currently being delivered in National Health Service (NHS) perinatal services in England. However, the evidence of its effectiveness is unclear. Exploring the current evidence of Gilbert’s CFT (13, 14) and Cree’s P-CFT (10, 11) for mothers in the perinatal period, Millard and Wittkowski’s (21) review identified and reported on five studies that provided insight into the intervention’s effectiveness for PMH difficulties (22–26). Millard and Wittkowski (21) noted that significant improvements were observed in compassion-based outcomes (i.e., self-compassion, self-criticism/self-reassurance) from pre- to post P-CFT intervention. However, four of these studies (22–25) included mothers from non-clinical populations only. Therefore, the current evidence base is applicable only to perinatal mothers presenting with sub-clinical symptoms (21).

Since Millard and Wittkowski’s (21) review, another two evaluations of P-CFT groups in P-CMHTs were published: in both evaluations, service users experienced more moderate to severe PMH difficulties (27, 28). Service users presenting with a wide range of mental health difficulties were included, such as anxiety, posttraumatic stress disorder (PTSD), complex PTSD or birth trauma, obsessive compulsive disorder (OCD), personality disorders, and eating disorders. Both evaluations reported significant improvements across compassion-based outcomes, mood, and postpartum bonding, but no control groups were implemented. Although these two evaluations provided an insight into the potential benefits of P-CFT, the data were extracted retrospectively from self-reported outcomes in electronic health records. It is unclear from these evaluations if a further exploration into P-CFT is feasible in an NHS perinatal service. Given the limitations of the above-mentioned studies, a study using a stronger methodological rigour is required to explore the potential benefits of P-CFT. However, prior to a large-scale efficacy study, further exploratory studies are warranted (29). This multisite study aims to explore the feasibility and acceptability of the P-CFT groups based on Cree (10, 11) and the study design in perinatal mothers and birthing parents through three components. More specifically, the objectives are as follows:

1. to explore the feasibility of the study design through recruitment and retention rates and quality of data collection (Component 1, Component 2),
2. to determine the suitability of the outcomes and their respective measures (Component 1),
3. to assess the acceptability of the intervention through a qualitative process evaluation to identify factors associated with engagement and adherence to P-CFT (Component 3),
4. to conduct an exploration of P-CFT has on compassion-based outcomes (i.e., self-compassion, self-criticism), psychological well-being and parent-infant interactions (Component 1, Component 2, Component 3).

## Methods

### Trial status

Recruitment started in November 2023 and is ongoing until approximately March 2026.

### Design

**Figure 1:**
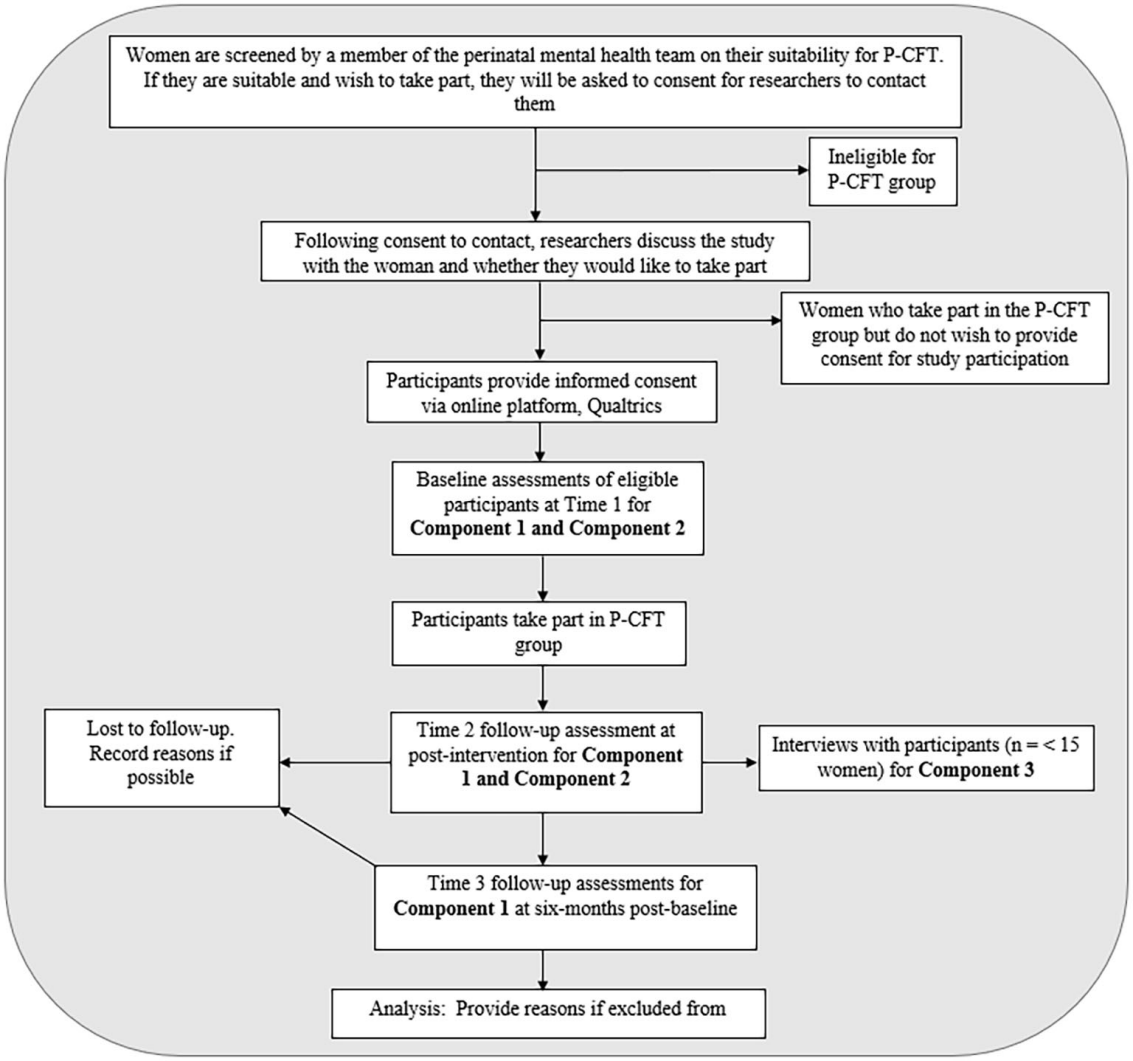
CONSORT diagram showing study design.

The study will adopt a single pre-post, quasi-experimental study design comprising three components (in this protocol, these components are referred to as Component 1, 2 and 3). This study design provides a convergent-parallel mixed methods process evaluation (30), which encapsulates the assessment of feasibility and acceptability, required as part of the MRC *Framework for Developing and Evaluating Complex Interventions* (29). The convergent-parallel approach entails the collection and analysis of quantitative and qualitative data, which are subsequently integrated and compared to allow for a unified analysis (30). Data from Components 1 and 2 will use quantitative methods and Component 3 will generate qualitative data. **Figure 1** displays an overview of the study design that reflects CONSORT guidelines (31), wherein a schedule of the study enrolment and assessments are provided in **Table 1**.

**Table 1:** Overview of recruitment and assessment timeframes.

Study Elements			Study period			
Component	Time point	Enrolment	Time 1 (Baseline)	Intervention (Week 1 to 10/12)	Time 2 (Post-intervention)	Time 3 (Follow-up)
Enrolment						
1, 2, 3	Eligibility screening	X				
1, 2, 3	Consent to contact	X				
1, 3	Informed consent		X		X	
Intervention						
1, 2, 3	P-CFT			X		
Assessments						
1	Demographic Questionnaire		X			
1	Fear of Self-Compassion Scale (FOSC) (32)		X		X	X
1	Sussex-Oxford Compassion for Self (SOCS) (33)		X		X	X
1	Postpartum Bonding Questionnaire (PBQ) (34)		X		X	X
1	Clinical Outcomes in Routine Evaluation (CORE-10) (35)		X		X	X
1	Depression, Anxiety, and Stress Scale-21 (DASS-21) (36)		X		X	X
1	The Short Warwick-Edinburgh Mental Well-Being Scale (SWEMWBS) (37)		X		X	X
2	Parent-infant video interaction		X		X	
3	Semi-structured interviews with up to 12–14 participants				X	

As part of Patient and Public Involvement and Engagement (PPIE), two groups from two of the study research sites were consulted on the project and design of the research. Points made by the PPIE groups were considered and incorporated into the study protocol and participant documents. Throughout the study, the PPIE group will be consulted as required, such as on dissemination processes.

### Setting

The feasibility and acceptability study will be conducted in NHS specialist perinatal community mental health teams (CMHT) that are offering group P-CFT as an intervention across three NHS trusts located in the northwest, southeast, and southwest of England. In England and Wales, specialist perinatal CMHTs offer multidisciplinary support to mothers, birthing parents, and their families who are experiencing moderate to severe mental health difficulties during pregnancy and at postpartum (8). Furthermore, they can offer treatment to mothers deemed at high-risk of postpartum relapse due to their histories of moderate and severe mental health conditions. This support includes psychological therapies, psychotropic medication, and parent-infant bonding support (8).

### Intervention and its delivery

The intervention is reported in line with the Template for Intervention Description and Replication (TIDieR) checklist (38), (see **Appendix A** in **Supplementary Materials** for further details). The group P-CFT intervention is based on Cree’s (11) adaptation of Gilbert’s (13, 14) CFT (see **Appendix B** for an overview of a P-CFT course). Whilst Cree’s (11) guidance is based on 12 sessions, the number of sessions can vary between services. In this study, the P-CFT intervention will range from eight to 12 sessions, which are to be delivered remotely via online video call platform Microsoft Teams (39) or in-person at a site of the perinatal CMHT. The two-hour sessions are offered once a week in consecutive weeks. There are two group facilitators in each group, and the number of participants can range from four to eight.

### Training and adherence to protocol checks

Of the two therapy facilitators in each P-CFT group, at least one of the facilitators will have received accredited training in the CFT and/or P-CFT model. Typically, the groups are run by two facilitators, who adhere to the CFT/P-CFT model and session content as outlined in their service P-CFT therapy manual, based on the core contents as listed in Cree’s (11) P-CFT adaptation (for further details see **Appendix B**). All group facilitators will be sent the study protocol and will attend a meeting with the lead researcher (LMB). This meeting will enable the facilitators to familiarise themselves with the study protocol. The lead researcher will be in regular contact with each of the research sites throughout the recruitment stage via phone and/or email communication to ensure protocol adherence.

### Component 1: feasibility study

#### Design

This component will use a non-randomised longitudinal, repeated measures design, across three research sites through routinely collected data and self-report outcome measures.

#### Participant inclusion/exclusion criteria

Mothers and birthing parents are first referred to and assessed by the P-CMHT to determine their suitability for the service. If they meet the service criteria, they can be referred to the P-CFT group. Once enrolled to receive P-CFT and consent-to-contact is provided, the research team will assess a service user’s eligibility against the study criteria. To participate in the study, it is required that mothers^1^ are at least 18 years of age and have a baby that is less than two years old at the time of starting the P-CFT group OR they are currently pregnant and can complete the intervention prior to approximately 34 weeks gestation. Participants are excluded from the study if they are not receiving care from a P-CMHT, deemed unsuitable by the P-CMHT to receive a group intervention, assessed as acutely at risk of harm to their baby, experiencing acute psychotic symptoms, alcohol or substance misuse, active suicidality and/or lacking capacity to consent under the Mental Capacity Act 2005 (41).

#### Recruitment and consent procedures

A member of the P-CMHT will identify potential eligible participants via case note review or in a multidisciplinary meeting. The member of the P-CMHT’s psychology team will then assess if they are suitable for the P-CFT group and if they can provide consent-to-contact regarding the study. If suitable, they will be sent a study leaflet and a copy of the participant information sheet from the lead researcher (LMB). Eligible service users have at least 24 hours to think about if they would like to take part. After 24 hours, the lead researcher will conduct a phone call with the potential participant. If the individual is happy to take part, they will be asked to provide their informed consent. All online forms will be hosted via Qualtrics (42). When providing informed consent, participants can also consent to Component 2 of this study concurrently, and opt-in to being contacted about Component 3. For Component 1, participants will complete a set of demographic questions, and six outcome measures (see **Table 1** for details). To reimburse and thank them for their time and contributions, participants can consent to being entered into a prize draw (ranging from a £25-£100 gift voucher) which will be conducted at the end of the study.

Study safety procedures will be outlined to participants as part of informed consent. Active monitoring of adverse events (AEs) will be conducted throughout the study with study participants and their clinical team. We will report any severe adverse events in a future publication reporting on outcomes.

#### Sample size consideration

Due to this study being a feasibility and acceptability study, formal sample size calculations were not undertaken. During the 28-month recruitment period of this study (from November 2023 to March 2026), it is estimated that approximately 17 groups will be offered across three NHS trusts (with ideally 4–8 participants in each group). Therefore, the potential pool of participants is 64 to 128 participants. It is planned to recruit 50% of this potential pool of participants (*n* = 32), approximately ten to 11 participants per site for baseline recruitment. This sample size reflects the median target baseline recruitment rate of 30 participants for feasibility studies in the United Kingdom (UK) (43). As an important part of the overall study aims to assess the feasibility of recruitment and retention, possible dropouts have not been factored into this calculation.

Retention of participants in the study will be examined using Lewis et al.’s (44) framework for assessing retention as a progression criterion. By applying the ‘traffic light system’, a retention rate above 85% will be considered acceptable (GREEN), 65% to 84% will be considered a borderline level of retention (AMBER), and below 65% (RED) will be deemed as a low level of retention. For any future studies on P-CFT in NHS perinatal services, a GREEN retention rate will indicate that the current study methodology is feasible for participant recruitment. Several amendments may be required to the methodology following an AMBER retention rate. A retention rate of RED demonstrates that considerable changes would be necessary to improve retention of participant recruitment. A preliminary exploration of attrition bias will also be conducted to examine if certain demographics or variables may influence attrition (e.g., number of children, financial situation).

#### Outcomes and assessments

For Component 1, participants will be asked to complete assessments at three time points. Baseline (or Time 1) assessments will be administered prior to the start of P-CFT (up to 4 weeks prior to the first treatment session). Post-intervention (or Time 2) assessments will be undertaken after the intervention phase (which will be up to 4 weeks post-intervention), with a final follow-up assessment at six months after baseline (referred to as Time 3).

Participants will be asked to complete a socio-demographic questionnaire which will provide information on the following: their NHS trust where they received P-CFT, current age, ethnicity, current relationship status, employment status, financial and socioeconomic situation, highest level of education, primary mental health diagnosis and whether or not they have received previous psychological treatment. The questionnaire will also collect data on age of youngest child and genders and ages of other children. At post-intervention, participants will be asked to provide the number of how many sessions of P-CFT they attended, as part of the online questionnaires.

#### Primary feasibility and acceptability outcomes

Data will be collected to indicate levels of feasibility and acceptability. Retention rates of participants in the study will be examined, which will be the number of consented participants who remain in Component 1 at post-intervention and follow-up. The number of eligible patients who consent to participate in the study as well as the number of patients who decline to participate will also be recorded to assess the feasibility of recruitment strategies. To assess the feasibility of the P-CFT intervention, the attendance of the group sessions will be collected. In addition, the rate of completion of the self-reported outcome measures across each time point will be used to signify their feasibility and acceptability.

#### Secondary clinical outcomes

A set of validated outcome measures will also be administered to assess the feasibility and acceptability of data collection in relation to compassion-based outcomes and mood. Details of the validated outcome measures are listed below and displayed in **Table 2**. Compassion will be examined using the *Fear of Compassion* (FCS) - *Fear of self-compassion subscale* (*FCS-SC*) (32) and the *Sussex-Oxford Compassion for Self scale (SOCS)* (33). This study will be able to assess if one compassion-based measure is more acceptable to participants. General presentation of mood will be measured with the *Clinical Outcomes in Routine Evaluation* (CORE-10) (35) and the *Depression, Anxiety and Stress Scale* (DASS-21) (36). The CORE-10 is a routinely collected measure in perinatal services (56). In addition to the DASS-21 use in other CFT studies, this measure was selected due to the associated improvements of anxiety and depression following CFT (18). Furthermore, the *Short Warwick-Edinburgh Mental Wellbeing Scale* (SWEMWBS) (37) will be administered to measure levels of wellbeing. Assessment of postpartum bonding will be evaluated using the *Postpartum Bonding Questionnaire* (PBQ) (34), which is also routinely collected in perinatal services (56). In addition, measures were selected based on their psychometric properties (see **Table 2** for details and the order of administration) and in consultation with the study’s PPIE group and the site group facilitators. As the CORE-10 and PBQ are measures that are already routinely collected by the perinatal services, participants are not required to complete these two measures as part of the study’s online questionnaire. In the event where consented participants do not complete the CORE-10 and PBQ during the study’s baseline assessment, the research team will request the scores from the group facilitators.

**Table 2:** Overview of the study’s outcome measures and psychometric properties in order of delivery.

Outcome measure properties									Psychometric properties	
Order of administration	Outcome measure	Aim	Item numbers	Scoring range	Score interpretation	Scale type	Response choices		Internal reliability (Cronbach’s alpha)	Test retest reliability
*1*	*Fear of Compassion* (FCS): *Fear of self-compassion subscale (*FCS-SC) (32)	Measuring kindness and compassion towards oneself	15 in total	0-21	Higher scores are indicative of increased fear of self-compassion	4-point Likert scale	‘Don’t agree at all’ to ‘Completely agree’		.92 (32)	Not available
*2*	*Sussex-Oxford Compassion for Self scale* (SOCS) (33)	Assesses how an individual relates to themselves	9 in total	0-21	Higher score reflects higher level of self-compassion	4-point Likert scale	‘Not at all’ to ‘Nearly every day’		.93 (45)	ICC:.73–78 (46)
*3*	*Postpartum Bonding Questionnaire* (PBQ) (34)	Measures difficulties in the parent-infant bond	25 in total Subscales: *12 for Impaired bonding (PBQ-IB)* *7 for Rejection and anger (PBQ-RA)* *4 for Infant-focused anxiety (PBQ-IFA)* *2 for Incipient abuse (PBQ-IA)*	0–125 total *PBQ-IB: 0-60* PBQ-RA: *0-35* *PBQ-IFA: 0–20 PBQ-IA:0-10*	Higher scores are indicative of increased bonding difficulties	6-point Likert scale	‘Always’ to ‘Never’		.78-.79 (47)	r = .77-.95 (48)
*4*	*Clinical Outcomes in Routine Evaluation-10* (CORE-10) (35)*	Assessing levels of psychological distress	10 in total	0-4	Higher scores are indicative of increased distress	5-point Likert scale	‘Not at all like me’ to ‘Extremely like me’		.82-.84 (49, 50)	r = .76 (51)
*5*	*Depression, Anxiety, and Stress Scale-21* (DASS-21) (36)	Measuring levels of depression, anxiety, and stress	0–21 in total. 7 for each subscale: *Depression (DASS-D)* *Anxiety 9DASS-A)* *Stress (DASS-S)*	0–21 for each subscale**	Higher scores reflect level of severity	4-point Likert scale	‘Never to ‘Almost always’		.76 -.93 (52)	ICC = .82 -.86 (53)
*6*	*Short Warwick-Edinburgh Mental Wellbeing Scale* (SWEMWBS) (37)	Assessment of well-being	7 in total	7-35	Higher scores are indicative of better well-being	5-point Likert scale	‘None of the time’ to ‘All of the time’		.88-.93 (54)	r = .86 (55)

* Measures that were routinely collected by the service.

**Summed score of each subscale is required to be multiplied by 2.

#### Data analysis

Data will be entered and then analysed using SPSS Statistics (57). Baseline characteristics and demographic characteristics will be tabulated. Prior to analysis, data will be checked in terms of errors in data entry, missing data, normality and outliers by the lead researcher (LMB). For missing data, intention-to-treat (ITT) analyses will be administered.

#### Data analysis of primary outcomes

The focus will be on tabulated and associated graphical summaries of the key indicators associated with the outcomes of a feasibility trial, which includes participant recruitment and attendance. Specifically, summary statistics will be the total number of referrals to the P-CFT groups, the number of service users who provide consent-to-contact and the number of service-users who provide their informed consent. Furthermore, group attendance and dropout rates, completion of follow-up outcome data and the number of participants who are retained at each timepoint will also be generated.

#### Data analysis of secondary outcomes

Summary statistics of the included measures (FCS-SC, SOCS, PBQ, CORE-10, DASS-21, SWEMWBS, CORE-10) at each timepoint will be presented in tables and/or graphs. Exploratory analyses of these outcomes will be conducted using the appropriate statistical method. The effect sizes generated from the secondary clinical outcomes, particularly the compassion-based measures, will also help to inform power analyses for larger scale definitive trials of P-CFT. To assess the effects of P-CFT at an individual-level, Jacobson and Truax’s (58) reliable change index will be utilised. For each measure, the percentage of participants that report a clinically significant change from Time 1 to Time 2 and Time 3 will be reported.

### Component 2: parent-infant interaction

#### Design

Component 2 will adopt a pre-post recorded observation of parent-infant interactions. Participants will be asked to make two short (six minute) video recordings of interactions between themselves and their infant, one prior to their participation in the CFT group, and one after the group finished.

#### Participant inclusion/exclusion criteria

The inclusion and exclusion criteria applied to the overall study is largely consistent across Component 1 and Component 2. However, participants who are pregnant at baseline (Time 1) are excluded from Component 2, because a recording of the parent-infant dyad is required.

#### Sample size consideration

Of the approximate 32 participants that completing the baseline assessment of Component 1, it is hoped that 50-75% of this sample will also consent to participate in Component 2. Therefore, Component 2 aims to recruit 16–22 parent-infant dyads, with ideally 5–8 participants collected from each research site.

#### Recruitment and consent procedures

Informed consent for Component 2 will be obtained prior to participation in Study 1. Once a participant has completed the baseline assessments of Component 1, the lead researcher (LMB) will contact participants who have consented to Component 2. All participants will be sent directions about setting up the space for the recording, which will be conducted either via videoconference call or as a self-recording by the participant. This method of remote data collection has been shown to be a feasible approach for caregivers (59).

#### Outcomes

##### Assessment of the parent-infant relationship

Videotaped parent-infant interaction will be rated for its global qualities using the *Manchester Assessment of Caregiver-Infant Interaction* (MACI-Infant) (60), a validated macroanalytic coding scheme. Coded videos of interaction are the “gold-standard” approach to assessing parent-infant relationship quality (61) and was included as a parent-infant attachment tool in a recent review of 25 measures by Shone et al. (62). MACI-Infant shows excellent stability over time, especially in maternal behaviours (60). The measure has been used in a range of populations including in mothers with mental health difficulties (63). MACI outcomes correlate with maternal and infant neural patterns (64–66), and empathy-specific mother-foetal attachment (67). MACI outcomes are also sensitive to change in interventions focused on enhancing the parent-infant relationship in other groups [e.g., (68)]. This assessment will allow us to examine whether the P-CFT group is associated with any enhancements in key parent-infant relational qualities. The MACI-Infant is suitable for infants aged 3 to 15 months old and consists of eight subscales, which are: 1) Caregiver sensitive responsiveness, 2) Caregiver non-directiveness, 3) Infant attentiveness to caregiver, 4) Infant positive affect, 5) Infant negative affect, 6) Infant liveliness, 7) Dyad mutuality, 8) Engagement intensity, and 9) Psychological stimulation. Each subscale is rated on a scale between 1 to 7. A score of 1 reflects a low frequency of a particular behaviour, and a score of 7 is indicative of a high frequency of a particular behaviour.

#### Procedure

Similarly to Component 1, the baseline recording for Component 2 will take place up to 4 weeks prior to the first treatment session (Time 1). Post-intervention (or Time 2) assessments will be undertaken after the intervention phase (which will be up to 4 weeks post-baseline assessments). Interactions will be recorded by either a remote call via secure videoconferencing [i.e., Microsoft Teams; (33)] or for participants to record the video using their own recording device. At the beginning of the call, the lead researcher (LMB) will guide the participant through the steps of the set-up. This set-up will involve setting up the computer/tablet/phone so that it is pointed to a corner of the room where the parent has brought some simple toys. The lead researcher will then record the interaction on the teleconferencing software and turn off their camera so that they are not visible to the participant for the duration of the play. For participants who self-record the interactions, participants will be asked to record only one six-minute interaction and send the lead researcher the video electronically.

#### Coding and data analysis

By a trained researcher, the parent-infant interaction videos will be coded using the validated coding scheme, MACI-Infant. Researchers trained in the coding scheme (MWW) will complete coding, and a random selection of 50% of coded material will be double coded by a researcher to ensure reliability and accuracy. Researchers will be supervised by trained, research quality-compliant coders. Typically, each six-minute recording undergoes at least two reviews, with pauses to document the observation and initial ratings in conjunction with the manual. Subsequently, a final review of the interaction is conducted to establish the ratings. The parent-infant interaction ratings from pre- and post-intervention will be compared using an appropriate statistical test selected at the time of data analysis.

### Component 3: qualitative research design

After the completion of the intervention, participants will be invited to take part in semi-structured interviews. Participants will be asked questions about the CFT group content, the group process, their ability and ease in showing compassion to self and others and how showing compassion might make an impact (if any) on their well-being, functioning and relationships with family, including their infant. Participants will be interviewed between one week and one-year post-intervention, which reflects similar previous studies (69).

#### Participant inclusion/exclusion criteria

The inclusion and exclusion criteria are replicated from the previous two studies in this protocol. Participants do not have to have taken part in Component 1 and 2 to be eligible for Component 3.

#### Sample size consideration

Across each NHS trust, the target sample size for the interview component of the study will be 12 (with a range of 10-15). This size of sample reflects other qualitative studies with similar aims and methods [e.g., (69)]. The aim would be to recruit around four participants from each research site.

#### Recruitment and consent procedures

There are two pathways for recruitment. If a participant provided initial consent for Component 1 to be contacted in relation to taking part in an interview, these participants will be contacted by the lead researcher (LMB) at the post-intervention stage. The second pathway to recruitment is that the study sites will identify potential participants who have completed at least 50% of a P-CFT group within the last 12 months in their service. Consent will be provided via written or oral (audio-recorded) communication. Following consent, participants will conduct a semi-structured interview with the main researcher (LMB) via Microsoft Teams (33). Furthermore, they will be asked to recall the number of P-CFT sessions they attended, which trust they received their P-CFT treatment, and how much time has passed since they attended the P-CFT group.

Demographic data will be collected following informed consent. If the participant has not completed Component 1 and Component 2 or there are any missing data, they will be asked to complete the set of demographic questions as listed in Component 1.

#### Interview topic guide development

Following feedback from PPIE, the topic guide was developed collaboratively between the authors (LMB, AW, DS). The topic guide aims to capture the participants’ reflections on their experiences of P-CFT through the following themes: being referred for a P-CFT group, the content and delivery of the P-CFT group, and whether there are any potential benefits from P-CFT.

#### Data analysis

Transcripts from the interviews will be generated by Microsoft Teams (39). For accuracy, a member of the research team (LMB) will check and revise the transcript where appropriate. Qualitative data will be analysed using reflexive thematic analysis [RTA; (70)]. This method has been selected for its potential to offer rich and detailed insights into real-world problems and for its theoretical flexibility, which is advantageous when exploring an under-researched area (70). The analysis will take an inductive, data-driven approach to enable as broad a range of potential themes and perspectives to emerge.

#### Reflexivity statement

The team have a diverse range of clinical and academic experiences regarding perinatal service users and qualitative research. The study forms part of the main researcher’s (LMB) PhD programme, who has experience conducting perinatal mental health and parenting research and working with service users in NHS mental health services. Specifically, three of the researchers involved in the study, who are all mothers, have expertise in health psychology and qualitative research (DS), developmental (MWW), and clinical psychology (AW) with substantial experience in perinatal mental health research. Specifically, AW is a qualified clinical psychologist and trained in P-CFT. Although the research team bring a breadth of knowledge to this study, this clinical and academic expertise may affect the interpretation of participants’ experiences. To safeguard potential biases, regular reflective discussions will take place among the author team when collecting and analysing the data.

## Discussion

To the authors’ knowledge, this study will be the first multi-site feasibility and acceptability study of perinatal CFT in specialist perinatal mental health services in NHS England. In general, the study will be able to explore and report if an online delivery of this intervention is beneficial for mothers and birthing parents in a specialist perinatal community mental health team. Specifically, the findings can help identify how the content and/or delivery can be adapted to optimise outcomes for P-CFT attendees. For instance, data collection of attendance levels per session will enable an objective measure of acceptability. An increase in attrition may indicate low acceptability of the content of one of the sessions. Possible observations such as this will be able to be further explored qualitatively. Moreover, service users within these perinatal clinical settings present with a range of mental health difficulties. Through the qualitative data collection, the suitability of P-CFT can be explored trans-diagnostically, allowing for further refinement of service delivery.

The suitability of recruitment methods can also be established to identify the type of recruitment methods that are appropriate for this specific type of intervention and clinical setting. Any potential barriers will be noted. For instance, one question is whether or not the recruitment strategies enable a sufficient sample size to be collected. Exploring potential barriers to recruitment and retention will highlight the type of practical issues that may need to be addressed when conducting any futures studies on P-CFT within a specialist perinatal mental health team for both service-users, staff members, and researchers.

In particular, the feasibility of the online implementation of the MACI, the parent-infant video interactions can be evaluated. In previous studies, the data collection of this tool has primarily been conducted in-person with the participants (60). However, this method was not feasible for this study due to the locations of the recruitment sites. As recruitment is being delivered online, some novel aspects require evaluation for feasibility. Srikar et al. (59) reported that an online/remote version of the MACI-Infant measure was feasible to use in mothers with infants at elevated likelihood of autism. Nevertheless, it is unclear if an online/remote delivery of the MACI-Infant is feasible among mothers with perinatal mental health difficulties. From a practical perspective, this current study will look at aspects such as whether or not the interaction can be sufficiently set up remotely by participants, whether the self-recordings can be correctly recorded and sent to the researchers through their home devices and the suitability of recording through teleconferencing software, and generally, whether this method is acceptable among the group of mothers and birthing parents. As Component 2 is an optional aspect of the study, the recruitment rate will be able to indicate the willingness of participants to take part in a caregiver-infant interaction study. A meta-synthesis of parental experiences of video feedback parenting interventions highlighted that participating in recorded video interactions can often elicit feelings of self-doubt and anxiety (71). The implementation of remote data collection may help to mitigate this potential issue.

It is important to identify the appropriate outcome measures for perinatal mothers and birthing parents experiencing mental health difficulties. Some of the outcome measures and/or their specific items may elicit distress in participants. By allowing the participants to skip items that they may not be comfortable in answering, the study can determine which specific items or questionnaires are acceptable in this population.

## Data Availability

The original contributions presented in the study are included in the article/**Supplementary Material**. Further inquiries can be directed to the corresponding author.

## Footnotes

1 In accordance with Darwin et al. (40) and for simplicity, the term ‘mother’ also includes birthing parents who identify as transgender or non-binary.
