# Supplementary material for: Perinatal compassion focused therapy for mothers with mental health difficulties: a study protocol for a multisite and mixed methods feasibility and acceptability study
Source: Front Psychiatry. 2025 Dec 11;16:1681673. doi: 10.3389/fpsyt.2025.1681673 (PMC12738376; doi:10.3389/fpsyt.2025.1681673)
Supplement: Supplementary file 1 [file DataSheet1.docx]

**Appendix A: The TIDieR (Template for Intervention Description and Replication) Checklist^32^**

| **Item number** | | **Item** | **Location of information** |
| --- | --- | --- | --- |
|  | **BRIEF NAME** | |  |
| **1.** | Provide the name or a phrase that describes the intervention. | | Pages 4-5, Appendix B |
|  | **WHY** | |  |
| **2.** | Describe any rationale, theory, or goal of the elements essential to the intervention. | | Pages 4-6, Appendix B |
|  | **WHAT** | |  |
| **3.** | Materials: Describe any physical or informational materials used in the intervention, including those provided to participants or used in intervention delivery or in training of intervention providers. Provide information on where the materials can be accessed (e.g. online appendix, URL). | | Page 8 |
| **4.** | Procedures: Describe each of the procedures, activities, and/or processes used in the intervention, including any enabling or support activities. | | Appendix B |
|  | **WHO PROVIDED** | |  |
| **5.** | For each category of intervention provider (e.g. psychologist, nursing assistant), describe their expertise, background and any specific training given. | | Page 8 |
|  | **HOW** | |  |
| **6.** | Describe the modes of delivery (e.g. face-to-face or by some other mechanism, such as internet or telephone) of the intervention and whether it was provided individually or in a group. | | Page 8 |
|  | **WHERE** | |  |
| **7.** | Describe the type(s) of location(s) where the intervention occurred, including any necessary infrastructure or relevant features. | | Pages 7-8 |
|  | **WHEN and HOW MUCH** | |  |
| **8.** | Describe the number of times the intervention was delivered and over what period of time including the number of sessions, their schedule, and their duration, intensity or dose. | | Page 8 |
|  | **TAILORING** | |  |
| **9.** | If the intervention was planned to be personalised, titrated or adapted, then describe what, why, when, and how. | | Page 8 |
|  | **MODIFICATIONS** | |  |
| **10.** | If the intervention was modified during the course of the study, describe the changes (what, why, when, and how). | | N/A |
|  | **HOW WELL** | |  |
| **11.** | Planned: If intervention adherence or fidelity was assessed, describe how and by whom, and if any strategies were used to maintain or improve fidelity, describe them. | | Page 8 |
| **12.** | Actual: If intervention adherence or fidelity was assessed, describe the extent to which the intervention was delivered as planned. | | N/A |

| **Session**  Appendix A: An example overview of a 12-week P-CFT programme | **Theme** |  | **Content** | **At-home exercises** |
| --- | --- | --- | --- | --- |
| 1 | **Introduction and the brain** |  | Psychoeducation; Our brain: Old and new | Soothing rhythm breathing |
| 2 | **Motives and emotions** |  | Understanding our emotions; mindfulness and new motherhood | Mindfulness |
| 3 | **The three systems** |  | The role of the threat, drive and soothing systems | Preparing the compassionate mind – The 5 stepping stones exercise |
| 4 | **What is compassion?** |  | Definition of compassion: skills and attributes | Safe place imagery |
| 5 | **Soothing system and imagery** |  | Thoughts and images: how they affect the mind and brain | Compassionate self; Compassionate colour |
| 6 | **Shame and compassion** |  | Shame and parenthood, compassionate imagery | Compassionate coach |
| 7 | **Compassionate attention** |  | The spotlight of attention | Mindfulness; Gratitude journal |
| 8 | **Individual session** |  | Formulation | Formulation diagram |
| 9 | **Compassionate thinking** |  | Reevaluating thoughts using the compassionate mind | Compassionate thoughts |
| 10 | **Compassionate letter writing** |  | Writing a compassion letter to oneself | Compassionate letter writing: Writing to your baby |
| 11 | **Compassionate behaviour** |  | Bringing compassion to the self-critic | Compassionate diaries |
| 12 | **Overcoming obstacles** |  | Summary of main topics; Navigating obstacles | End of therapy |

**Appendix B: An example overview of a 12-week P-CFT programme**
